# Elevated levels of vitamin D and deficiency of mannose binding lectin in dengue hemorrhagic fever

**DOI:** 10.1186/1743-422X-9-86

**Published:** 2012-05-04

**Authors:** Kalichamy Alagarasu, Rupali V Bachal, Asha B Bhagat, Paresh S Shah, Cecilia Dayaraj

**Affiliations:** 1 Dengue group, National Institute of Virology, 20-A, Ambedkar road, Pune, Maharashtra, India; 2 National Institute of Virology, 20-A, Ambedkar road, Pune 411001, Maharashtra, India

**Keywords:** Dengue, Vitamin D, Mannose binding lectin, DF, DHF

## Abstract

**Background:**

Altered plasma concentrations of vitamin D and mannose binding lectin (MBL), components of innate immunity, have been shown to be associated with the pathogenesis of viral infections. The objective of the present study was to find out whether plasma concentrations of MBL and vitamin D are different in patients with dengue fever (DF) and dengue hemorrhagic fever (DHF).

**The results:**

The plasma concentrations of vitamin D and MBL were assessed in 48 DF cases, 45 DHF cases and 20 apparently healthy controls using ELISA based methods. Vitamin D concentrations were found to be higher among both DF and DHF cases as compared to healthy controls (*P* < 0.005 and *P* < 0.001). Vitamin D concentrations were not different between DF and DHF cases. When the dengue cases were classified into primary and secondary infections, secondary DHF cases had significantly higher concentrations of vitamin D as compared to secondary DF cases (*P* < 0.050). MBL concentrations were not significantly different between healthy controls and dengue cases. MBL concentrations were observed to be lower in DHF cases as compared to DF cases (*P* < 0.050). Although MBL levels were not different DF and DHF cases based on immune status, the percentage of primary DHF cases (50%) having MBL levels lower than 500 ng/ml were less compared to primary DF cases (*P* = 0.038).

**Conclusions:**

The present study suggests that higher concentrations of vitamin D might be associated with secondary DHF while deficiency of MBL may be associated with primary DHF.

## Background

Dengue, caused by dengue virus (DENV), constitutes a public health emergency of international concern. DENV infection in humans results in a spectrum of outcomes ranging from asymptomatic to undifferentiated fever, mild form of the disease namely dengue fever (DF) to severe forms including dengue hemorrhagic fever (DHF) and dengue shock syndrome (DSS) that may be fatal [1]. The outcome of DENV infection is determined by multiple factors including viral virulence, host genetics and host immune responses [2].

Among the various components of host immune responses, T cells, antibodies, cytokine storm and complement factors contribute to the pathogenesis of dengue [2]. Epidemiological studies have shown an association between DHF/DSS and secondary DENV infection. Preexisting antibodies and cross reactive T cell responses induced by the primary infection is believed to exacerbate the disease during secondary infection [3,4]. Proinflammatory cytokines namely interleukin-8 (IL-8), tumor necrosis factor-α and interferon-γ and anti inflammatory cytokine IL-10 also contribute to dengue disease pathogenesis [5-11]. Activation of T cells, antibodies and cytokines are influenced by various immunomodulators. Increase or decrease in the levels of these immunomodulators influences the outcome of viral infections [12].

Vitamin D is a potent immunomodulator affecting both innate and adaptive immune responses. Vitamin D binds to Vitamin D receptor (VDR), translocates to the nucleus and influences gene expression. Vitamin D enhances the phagocytic capacity of macrophages and induces antimicrobial peptide gene expression contributing to innate immune responses [13]. Vitamin D inhibits T-helper 1 (Th1) cell and cytotoxic T cell responses. It decreases B-cell proliferation, plasma-cell differentiation and IgG secretion [14]. Vitamin D also enhances Th2 cytokine and IL-10 responses [15,16]. Vitamin D deficiency increases the risk of cancer, tuberculosis, as well as influenza and human immunodeficiency virus infection [12,17,18]. Vitamin D has been reported to influence the expression of DENV receptors in immune cells [19-21]. A study from Vietnam has shown the association of vitamin D receptor gene polymorphisms with susceptibility to DHF [22].

One of the major pathways of complement activation is initiated by binding of the virus to mannose binding lectin (MBL). MBL is a pattern recognition molecule that recognizes specific sugar molecules present on the surface of microorganisms including DENV [23,24]. Point mutations in the *MBL2* gene lead to reduced concentrations of functional oligomers. Genetically determined variation in serum concentrations of MBL has been shown to influence the susceptibility to infectious, autoimmune and cardiovascular diseases [23]. Alleles of *MBL2* gene that are associated with higher concentrations of functional MBL, have been shown to be associated with thrombocytopenia in dengue infected patients [25]. MBL concentrations were also found to be increased in acute samples of DHF cases as compared to DF cases [26].

Since MBL and Vitamin D are known to influence innate and adaptive immune responses and DENV pathogenesis is immune mediated, we hypothesized that altered levels of plasma vitamin D and MBL might be associated with dengue disease severity. Therefore, we investigated the levels of plasma vitamin D and MBL in dengue infected patients in the context of disease severity and immune status.

## Results

### Demographic and clinical characteristics of patients

Among the 93 patients included in the study, based on the DF/DHF defining criteria of the World Health Organization (WHO) [27], 48 had DF and 45 had DHF. Males were over represented in both DF and DHF patients. The male to female ratio in DF was 2:0.76 and in DHF, it was 2:1. Demographic and clinical characteristics of patients were given in Table 1. The median age of DHF cases (23.0 years) was significantly lower than that of DF cases (29.5 years) (*P* = 0.034). The number of primary cases with DF (42.5%) was higher than the number of primary cases with DHF (18.6%) (*P* = 0.026). When the presence of clinical symptoms was compared between DF and DHF cases, the presence of fever with chills, headache, myalgia, arthralgia, retro orbital pain and rash were reported equally in DF and DHF cases. Presence of nausea/vomiting and abdominal pain was represented by the DHF cases. Thrombocytopenia was significantly over represented in DHF cases as compared to DF cases (*P* < 0.001). The median count of platelets was significantly lower in DHF cases (*P* = 0.020) (Table 1).

**Table 1:** Demographic and clinical characteristics of dengue patients

**Clinical and demographic characteristics**	**DF cases n = 48 (%)**	**DHF cases n =45 (%)**	***P* value**
Age*	29.5 (11–65)	23.0 (4–58)	0.026
Immune status**	20 Primary (42.5) & 27 secondary (57.4.)	8 primary (18.6) & 35 secondary (81.3)	0.040
Fever	48 (100.0)	45 (100.0)	1.000
Headache	24 (50.0)	23 (51.1)	0.920
Myalgia	28 (58.3)	22 (48.9)	0.480
Arthralgia	7 (14.6)	9 (20.0)	0.670
Nausea/Vomitting	0	12 (26.7)	<0.001
Abdominal pain	0	5 (11.1)	0.023
Retro orbital pain	1 (2.1)	4 (8.9)	0.190
Rash	9 (18.8)	9 (20.0)	0.950
Thrombocytopenia	28 (58.3)	41 (91.1)	<0.001
Platelet count*	67000 (13000–330000)	38500 (5000–150000)	0.002

*Age and platelet counts are represented in the form of median values with ranges in the parenthesis and compared between DF and DHF cases using Mann–Whitney *U* test.

**For determining immune status, only 47 DF and 43 DHF samples were available.

Among the 45 DHF cases, gastrointestinal bleeding, manifested by melena or hematemesis was reported in 21 (46.7%) cases, hematuria was observed in six (13.3%), gum bleeding in seven (15.6%), conjunctival hemorrhage in one (2.2%) and epistaxis in one (2.2%). Plasma leakage was observed in 9 (20%) patients either as ascites (n = 5) and/or as pleural effusion (n = 4). Shock/hypotension was observed in five patients (11.1%). No fatality was observed in the cases included in the study.

### Plasma concentrations of vitamin D in dengue patients

**Figure 1:**
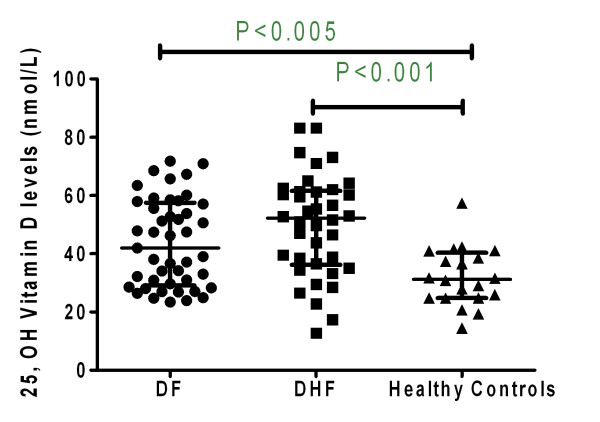
25-hydroxy vitamin D concentrations in DF cases, DHF cases and healthy controls. 25-hydroxy vitamin D concentrations were expressed in ng/ml and plotted in form of a scatter plot. The lines represent median value with interquartile ranges. The number of samples for DF is 45, 38 for DHF and 20 for healthy controls.

Plasma 25-hydroxy vitamin D_3_ (vitamin D) concentrations were investigated in 45 DF cases, 38 DHF cases and 20 healthy controls. Vitamin D concentrations were found to be significantly higher in DF and DHF cases as compared to healthy controls (healthy controls vs. DF cases *P* < 0.005; healthy controls vs. DHF cases *P* < 0.001). Among DF and DHF cases, the vitamin D concentrations were found to be higher in DHF cases, though, the difference was not statistically significant (*P* > 0.050) (Figure 1). The sample size has a power of 0.94 and 0.80 to detect the differences observed between DHF and healthy controls and between DF and healthy controls respectively.

### Vitamin D levels in the context of immune status and disease severity

**Figure 2:**
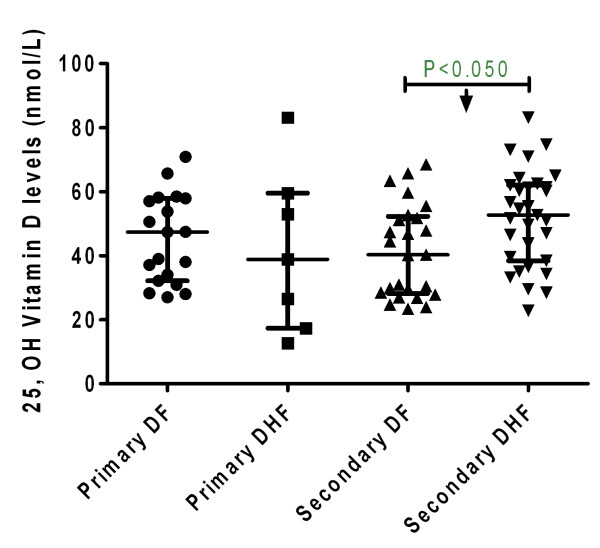
25-hydroxy vitamin D concentrations in DF cases and DHF cases based on the host immune status. 25-hydroxy vitamin D concentrations were expressed in ng/ml and plotted in form of a scatter plot. The lines represent median value with interquartile ranges. The number of samples for primary DF is 19, 7 for primary DHF, 25 for secondary DF and 31 for secondary DHF.

When the patients were grouped based on immune status and disease severity, secondary DHF cases had significantly higher concentrations of vitamin D as compared to secondary DF cases (*P* < 0.050). The sample size has a power of 0.79 to detect the differences observed between secondary DF and secondary DHF cases. Vitamin D concentrations were not significantly different between primary DF and primary DHF cases (*P* > 0.050). When comparisons were made between primary DF and Secondary DF or primary DHF and secondary DHF, vitamin D levels were not different (*P* > 0.050) (Figure 2).

### Plasma MBL concentrations in dengue patients

**Figure 3:**
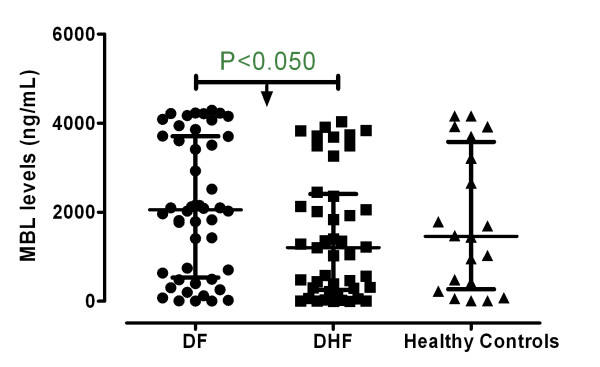
Mannose binding lectin levels in DF cases, DHF cases and healthy controls. Mannose binding lectin levels were expressed in ng/ml and plotted in form of a scatter plot. The lines represent median value with interquartile ranges. The number of samples for DF is 48, 45 for DHF and 20 for healthy controls.

Plasma MBL concentrations were assessed in 48 DF cases, 45 DHF cases and 20 healthy controls. MBL concentrations were not significantly different between healthy controls and DF or DHF cases (*P* > 0.050). When DF and DHF cases were compared, significantly lower concentrations of MBL were observed in DHF cases (*P* < 0.050) (Figure 3). The sample size has a power of 0.52 to detect the observed differences between DHF and DF cases. The sample size is underpowered to detect the differences (effect size of less than 0.5) observed between DF and healthy controls or DHF and healthy controls. However, the sample size has a power of above 0.80 to detect an effect size of 0.8 and above.

### Plasma MBL concentrations in the context of immune status and disease severity

**Figure 4:**
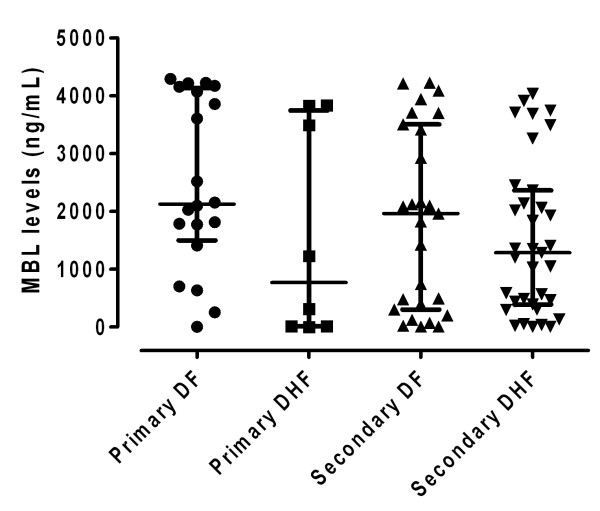
Mannose binding lectin levels in DF and DHF cases based on the host immune status. Mannose binding lectin levels were expressed in ng/ml and plotted in form of a scatter plot. The lines represent median value with interquartile ranges. The number of samples for primary DF is 20, 8 for primary DHF, 27 for secondary DF and 35 for secondary DHF.

When the patients were classified into primary and secondary cases and compared, irrespective of DF or DHF, MBL concentrations were not different (*P* > 0.050). MBL concentrations were lower in primary DHF cases as compared to primary DF cases though not statistically significant. MBL levels were not different between secondary DF and secondary DHF (Figure 4).

### Categorization of dengue patients based on MBL deficiency

Since MBL level varies as a function of multiple polymorphisms of *MBL2* gene, a cutoff value of 500 ng/ml was used to define subjects with or without MBL deficiency. Based on the cutoff value, 40% of DHF cases, 25% of DF cases and 35% of the healthy controls had deficiency of MBL. When the patients were grouped based on disease severity and immune status, 50% of primary DHF cases had MBL deficiency as compared to 10% of primary DF cases (*p* = 0.038, Odds ratio (OR) 9, 95% confidence limits 0.84–120). This difference was not observed in secondary infections with 37% of secondary DF and 40% of secondary DHF patients having low level of MBL. Analysis with a cutoff value indicating almost complete deficiency of MBL (<100 ng/ml) was also performed. Results revealed that 37.5% of primary DHF cases had an almost complete deficiency of MBL as compared to 5% of primary DF cases (*p* = 0.058, OR 11.40 95% CI 0.66–628). 14.8% of secondary DF and 14.2% of secondary DHF patients had an almost complete deficiency of MBL.

## Discussion

In the present study, plasma concentrations of vitamin D and MBL were investigated in dengue patients from Pune, Maharashtra, western India. Pune is endemic to dengue with 600–800 cases occurring annually. All four serotypes have been reported to be circulating in Pune [28]. The demographic and clinical characteristics reported in the present study were similar to that reported in our earlier study [5].

Investigation of vitamin D concentrations revealed significantly higher concentrations of vitamin D in dengue patients (both DF and DHF) as compared to apparently healthy controls suggesting that higher concentrations of vitamin D might be associated with symptomatic disease. Further analysis revealed that the association was more evident in secondary DHF. It has been shown that vitamin D induces expression of dendritic cell specific intercellular adhesion molecule 3 grabbing non integrin (DC-SIGN), the primary receptor for DENV entry into immature dendritic cells [19]. It has also been shown that calcitriol (active form of vitamin D) increases the expression of Fcγ receptors on human monocytic cell lines and monocyte derived dendritic cells [20,21]. The increased concentrations of vitamin D, in DENV infected cases with secondary infection, might enhance viral entry through increased expression of Fcγ receptors leading to higher viral load, uncontrolled inflammatory responses and subsequent development of DHF. Vitamin D is also known to suppress Th1 cytokines and enhance IL-10 production by peripheral blood mononuclear cells in response to microbial antigens [14-16]. Since IL-10 is known to play a role in dengue disease pathogenesis [11], it is possible that vitamin D could also contribute to disease pathogenesis through altered IL-10 response. Since the effect of vitamin D is also dependent on single nucleotide polymorphisms in the VDR gene [29], the influence of vitamin D on dengue in the context of host genetics needs to be investigated.

Analysis of circulating concentrations of MBL in dengue cases and healthy controls revealed no significant difference between the two groups suggesting that the MBL mediated pathway of complement activation might be inhibited or may not be induced during DENV infection. Deficiency of MBL has been reported in patients infected with other RNA viruses such as Crimean-Congo hemorrhagic fever virus, respiratory syncytial virus and severe acute respiratory syndrome (SARS) corona virus [30-32]. Interaction between complement components and non structural protein 1 (NS1) of flaviviruses has been reported to inhibit classical and lectin pathways of complement activation [33]. The present study revealed significantly lower levels of MBL in DHF cases suggesting that reduced activation of MBL mediated complement pathway might be associated with DHF. MBL is known to interact with N- linked glycans of structural proteins of DENV and neutralize the virus by blocking viral fusion. In experimentally infected mice, MBL dependent intravascular clearance of DENV has also been reported [24]. Therefore, it is possible that MBL deficiency might have led to decreased activation of MBL mediated pathway of complement and reduced intravascular clearance of DENV leading to higher viral load. Higher viral load has been shown to be associated with DHF in several studies [34,35].

In the present study, deficiency of MBL was more evident in cases with primary DHF. The association of MBL deficiency with DHF in primary infection also suggests that the protective effects of MBL and the innate immune responses are more important during primary infection which could be overshadowed by presence of cross reactive complement fixing antibodies during secondary infection [36]. In contrast to the present study, a study from Brazil has reported higher concentrations of MBL among DHF cases [7]. A higher MBL concentration might also lead to increased inflammation through enhancement of the production of pro-inflammatory cytokines. Increased concentration of factor D and decreased concentration of factor H have been reported in DHF cases suggesting that imbalance in the regulation of factors H and D of the alternative pathway of complement activation is associated with DHF [26]. Since MBL concentrations are dependent on the presence of mutations in the structural and promoter regions of *MBL2* gene, it is possible that variant alleles of *MBL2* gene polymorphisms might be associated with DHF. A case control study from Brazil has shown the association of wild type alleles of the MBL2 gene with thrombocytopenia in dengue patients [25]. Further case control studies are needed to confirm the phenotypic effects of *MBL2* gene polymorphisms in dengue patients with primary infection from India. Although, the present study is sufficiently powered to detect intermediate to large effect size, with regard to MBL, the study is underpowered and further studies with larger sample size are needed to confirm the preliminary associations.

## Conclusions

The present study suggests that higher concentrations of vitamin D are associated with secondary DHF. This association may be related to the inducing effect of vitamin D on Fcγ receptor expression which might subsequently lead to higher viral load in dengue cases with secondary infection and hence development of DHF. The study also suggests that MBL deficiency is associated with primary DHF. This association might be related to the reduced activation of the MBL pathway of complement leading to higher viral load in dengue cases with primary infection. This is the first study that correlates the concentrations of vitamin D and MBL with immune status of dengue cases.

## Subjects and Methods

### Clinical samples

Blood samples from patients with dengue like illness were referred to the National Institute of Virology for diagnosis. Samples were transported on ice and plasma was separated and aliquoted. One aliquot was used for dengue specific IgM ELISA and leftover aliquots were stored in -80°C. A total number of 93 samples, which were positive for dengue specific IgM ELISA, were included in the study. All the samples were collected during the seasonal outbreak in Pune in 2009. Clinical presentations of the patients recorded by the clinicians were used to classify the patients. The patients were classified into those with DF and those with DHF. Patients with fever, headache, myalgia, retro-orbital pain, and rash were defined as DF. DHF patients were categorized by the presence of at least two of the DHF defining criteria of the WHO [27]: hemorrhagic tendencies/manifestations, thrombocytopenia, and evidence of plasma leakage. Samples from 20 apparently healthy blood donors were also used in the study. This study was approved by the National Institute of Virology Human Ethics Committee. Waiver of the informed consent was granted by the committee on the basis of “Use of leftover specimens after clinical investigation” under the Indian Council of Medical Research Guidelines 2006.

### Laboratory diagnosis

The in-house National Institute of Virology (NIV) IgM capture ELISA kit was used to detect DENV-specific IgM. A known positive (P) and a known negative (N) serum control were used in every test. A sample showing P/N ratio >2.1 times the optical density was considered positive. The IgG capture ELISA (E-DEN02G, Panbio, Windsor, Australia) was used to classify the cases into primary or secondary DENV infection. IgG levels of >22 units (defined by the manufacturers) indicated secondary infection.

### Estimation of Vitamin D and MBL concentrations

25-hydroxyvitamin D_3_ (vitamin D) was quantitated in the plasma samples using an enzyme immunoassay kit (IDS Ltd, UK) according to the manufacturer’s protocol. Calibrators and controls were used in each assay. The percent binding (B/B%) of each calibrator, control and unknown samples were calculated by the following formula: B/B% = mean absorbance/mean absorbance for ‘0’ calibrator x100. A calibration curve with B/B% and vitamin D concentrations were used to find out the concentrations of vitamin D in unknown samples in nm/L. The detection limit of the kit is 5 nm/L.

Estimation of plasma MBL concentrations was done using the MBL oligomer ELISA kit (BioPorto Diagnostics, Denmark) according to the manufacturer’s instructions. Calibrators were used in each assay and a calibration curve was constructed by plotting the mean absorbance values for each calibrator on the y-axis against the corresponding MBL concentrations in ng/ml on the x-axis. The MBL concentration of each diluted plasma sample was then found by locating the point on the curve corresponding to the absorbance value of each diluted plasma sample and reading its corresponding concentration in ng/ml from the x-axis. The concentration of MBL in the undiluted plasma sample is calculated by multiplying this result by the sample dilution factor. The detection limit of the kit is 2 ng/ml.

### Statistical analysis

Using the Statcalc program (Epi info version 6.0.4, CDC, Atlanta, GA, July 1996), the chi-square test with Yates correction or Fisher exact test (when any cell value was less than 5) was performed to examine differences in demographic and clinical characteristics of the dengue patients. Age and platelet counts were compared using Mann–Whitney *U* test. Concentrations of MBL and 25[OH]D in plasma samples were compared between study groups using Kruskal-Wallis test with Dunn’s multiple comparison for selected groups. The *P* values from Dunn’s multiple comparison for selected groups were provided.

Since MBL levels varies depending on the presence of polymorphisms in the *MBL2* gene, categorization of study subjects based on MBL deficiency (defined by a cutoff value of <500 ng/ml) was done and compared between study groups using the chi-square test with Yates correction or Fisher exact test. This cutoff value (<500 ng/ml) has been earlier shown to be a reliable predictor of low producing MBL2 genotypes using receiver operating characteristic analysis with individual data from 1642 healthy subjects from 4 studies [37].

All statistical analysis was performed using Graphpad prism (version 4). A Two tailed *P* value less than 0.05 was considered significant. Power calculations were performed using the software G*Power version 3.1.3. The achieved power for significant results were calculated using the Wilcoxon-Mann–Whitney test (two groups) option available in ‘t’ test family of the software. The sample size, level of significance and effect size were provided as input. For calculating the effect size, the software uses mean values with standard deviation of the two groups [38].

## Abbreviations

DENV = Dengue virus; DF = Dengue fever; DHF = Dengue hemorrhagic fever; DSS = Dengue shock syndrome; IL = Interleukin; TNF = Tumour necrosis factor; IFN = Interferon; VDR = Vitamin D receptor; MBL = Mannose binding lectin; Vitamin D = 25-hydroxy vitamin D3; OR = Odds ratio; DC-SIGN = Dendritic cell specific intercellular adhesion molecule; Th = T helper; SARS = Severe acute respiratory syndrome; P = Positive; N = Negative.

## Competing interests

The authors declare that they have no competing interests

## Authors’ contributions

Conceived and designed the experiments: CD, KA & PSS. Performed the experiments: KA, RVB, ABB. Analyzed the data: KA. Coordinated the sample collection and diagnosis: PSS. Wrote the paper: KA, CD. All authors read and approved the final manuscript.
